# How Can We Provide Additively Manufactured Parts with a Fingerprint? A Review of Tagging Strategies in Additive Manufacturing

**DOI:** 10.3390/ma15010085

**Published:** 2021-12-23

**Authors:** Antonella Sola, Yilin Sai, Adrian Trinchi, Clement Chu, Shirley Shen, Shiping Chen

**Affiliations:** 1 Commonwealth Scientific and Industrial Research Organisation (CSIRO), Manufacturing Business Unit, Clayton, VIC 3169, Australia; adrian.trinchi@csiro.au (A.T.); clement.chu@csiro.au (C.C.); shirley.shen@csiro.au (S.S.); 2 Commonwealth Scientific and Industrial Research Organisation (CSIRO), Data61, Eveleigh, NSW 2015, Australia; yilin.sai@data61.csiro.au (Y.S.); shiping.chen@data61.csiro.au (S.C.)

**Keywords:** additive manufacturing, 3D printing, tag, traceability, provenance, anti-counterfeiting, authentication, identification

## Abstract

Additive manufacturing (AM) is rapidly evolving from “rapid prototyping” to “industrial production”. AM enables the fabrication of bespoke components with complicated geometries in the high-performance areas of aerospace, defence and biomedicine. Providing AM parts with a tagging feature that allows them to be identified like a fingerprint can be crucial for logistics, certification and anti-counterfeiting purposes. Whereas the implementation of an overarching strategy for the complete traceability of AM components downstream from designer to end user is, by nature, a cross-disciplinary task that involves legal, digital and technological issues, materials engineers are on the front line of research to understand what kind of tag is preferred for each kind of object and how existing materials and 3D printing hardware should be synergistically modified to create such tag. This review provides a critical analysis of the main requirements and properties of tagging features for authentication and identification of AM parts, of the strategies that have been put in place so far, and of the future challenges that are emerging to make these systems efficient and suitable for digitalisation. It is envisaged that this literature survey will help scientists and developers answer the challenging question: “How can we embed a tagging feature in an AM part?”.

## 1. Introduction

Additive manufacturing (AM), also known as 3D printing, is a very fast-growing field of research [1]. According to recent statistics, the market will accelerate at a rate of 14.4% from USD 8.35 billion in 2019 to USD 23.75 billion in 2027 with the manufacturing industry currently holding 35.6% of the market share [2]. With the progressive shift from “rapid prototyping” to “industrial production”, new needs are emerging to provide AM parts with tags or marks that, like a fingerprint, allow their origin to be recognised or to distinguish them from other, apparently similar, items. The traceability of AM parts is becoming critical in the cutting-edge areas of biomedicine (e.g., implants, scaffolds), aeronautics (e.g., jet engines, specialty parts), space (satellites, spacecraft) and defence, where the failure of 3D printed objects can result in catastrophic consequences and even pose a major threat to the safety of human beings [3,4]. Whereas several methods are already available for the traceability of food [5,6], textiles [7] and items produced by conventional manufacturing [8], it is not clear yet how such methods can be translated to AM. The main goal of this review is to outline how materials science and technology can contribute to bridging this gap and developing new strategies to embed provenance information into 3D printed objects. After presenting the main reasons driving research in certification of AM products, the subsequent sections describe the main approaches proposed in the literature for incorporating a tagging feature in 3D printed parts. Whereas the prevailing solution consists of printing a quick response (QR) code within the part, numerous other methods are available, which range from encasing a detector to leveraging the stochastic defects of AM parts. Wherever possible, information has been reported on the tagged object’s microstructure. As outlined in Section 5, many challenges are still open to make these tagging features more practical and to streamline their incorporation. Additional efforts are deemed necessary to understand how the presence of the tag will modify the microstructure and the mechanical performance of the printed part. In the future, prompted by the digitalisation of traceability information, tagging features are expected to become the link between physical and cyber worlds, and this calls for a deeper understanding of the printed object–tag–virtual twin integration.

## 2. Traceability and AM

### 2.1. Authentication or Identification?

Although this point is often blurred in the available literature on AM parts, a basic distinction should be drawn between “authentication” and “identification”. Authentication means to certify an item as being genuine through its origin from a specified brand/company, typically via a distinctive mark or feature. Identification means that an item is marked to pick it individually. According to the United States Pharmacopeia–National Formulary (USP–NF), enforcing the “integrity” implies “Ensuring the identity and authenticity of a material or product by a set of procedures” [9].

Quite often the branding may serve as a surrogate authenticator, as the company or product logo may contain unique design/artwork that alludes to it being the genuine article. However, authentication does not automatically imply identification, because all the items bearing the same authenticating logo could be shown as genuine, and the authenticating mark may not delineate different product categories from the same brand. 

Identifying markers can be used in several different ways such as to distinguish one brand from another (e.g., a trademarked company logo), to identify product ranges within a brand or to specify each individual manufactured product (e.g., a serial number), and they can even imply authentication. In fact, identification conveys more comprehensive information than authentication, since an identification mark can authenticate the origin and, additionally, allow each single item to be distinguished from all similar items coming from the same producer. In other words, whereas an authentication mark is not necessarily an identification mark, an identification mark often works also as an authentication mark.

A meaningful term of comparison is provided by cars. Usually, each car clearly exhibits a tag that identifies the manufacturer’s name or logo, along with additional markings to identify the particular vehicle model. It also displays a number plate that validates registration and ownership, thus distinguishing the vehicle from another one of the same series. When coupled with the model’s unique aesthetic, these features may appear to authenticate the vehicle. However, plates can be easily removed or replaced, either for malicious purposes or just by accident. Fortunately buried within the vehicle are an array of less obvious identifiers, such as engine numbers, compliance plates, vehicle identification numbers (VINs), and even more intricate hidden identifiers embedded within things, such as paint, which are difficult to reach and almost impossible to counterfeit or tamper. All of these, when analysed, serve to both identify and authenticate each individual vehicle.

### 2.2. Why Authenticate and Identify AM Parts?

AM has broad-ranging appeal, from hobbyists printing models, engineers manufacturing highly specialized components for aerospace applications, to medical professionals developing bespoke biomedical implants. Traditional product identification and branding methods, such as trademarks, which advertise and make clear that “that” item comes from “that” producer, may be suitable for consumer- and hobby-level parts, as they are not deemed critical, and they do not require or justify the expense of a higher degree of validation. 

**Figure 1:**
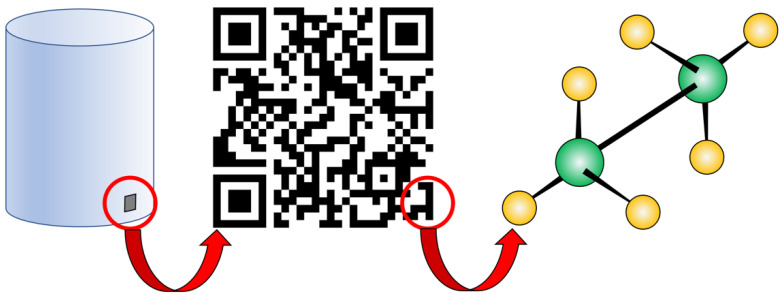
Example of a multi-level tagging feature that is composed of an ouverte QR code and of a chemical fingerprint (the static QR code redirects to A. Sola’s ORCID page, https://orcid.org/0000-0002-8649-9388 (accessed on 22 December 2021); the image is only for illustrative purposes, and the chemical structure shows a mock compound).

For more high-valued and critical parts, besides promoting the product’s brand, authentication also serves more critical functions. Unqualified parts not only undermine the quality and reliability of the associated devices they are installed in, but could also pose threats to human lives. Many devices in the aerospace, defence, automotive, and medical industries have safety-critical functions such as turbine blades in jet engines, structural components in vehicles, and biomedical implants. The unqualified use of AM parts could have disastrous consequences, and such parts may require authentication for liability reasons [4,10]. For instance, an AM bio-implant requires assurance that not only the part is genuine, but that it also has been identified as the correct part for the specific patient. Unique identification is pivotal to complying with traceability requirements and to avoiding counterfeiting of high end-value parts [11]. As stressed by Eisenbarth et al. [12], the problem is particularly serious for AM parts, since nearly any geometry can be (re-)produced by AM with stolen data or with reverse engineering of the original prototype, but the mechanical and functional properties of the fake part may be substantially different from the original ones. Even if the geometry is exactly the same, parts manufactured with a cheaper low-quality material or with non-optimised processing parameters will likely pass the visual inspection and preliminary quality checks but will experience premature failure in exercise [13]. As AM shifts from rapid prototyping to scale manufacturing and products begin to travel in a supply chain, distributers and end users will need to accomplish field validation [14]. In this case, authentication features are required that are more difficult to reproduce than merchandising logos. In addition, like a car that possesses a logo, plate number, and chassis serial number, a single object may receive multiple marks or multi-level tags that respond to different authentication and identification needs as illustrated in Figure 1.

Identification is also necessary to facilitate logistics, especially to manage and track the flow of items down the supply chain of large-scale production [11,15]. Interestingly, as an example of logistics that works on a small-length scale, Paz et al. [16] recently dedicated a contribution to the identification of AM medical instruments for real-time tracking and location during surgical interventions.

The scientific literature on this topic is still relatively limited. A bibliographic search conducted in Scopus on 24 October 2021, entering “additive manufacturing” AND “anti-counterfeiting” as keywords in “article title, abstract, keywords” returned only 17 results. Whereas the majority of these papers described polymer-based parts, the list narrowed down to six results upon adding “metal” as an additional keyword, with the search results including the experimental contributions by Eisenbarth et al. [12], Flank et al. [14], Terranova et al. [17], and Wei et al. [10], the review paper on 3D printable inks based on coordination compounds by Maldonado and Amo-Ochoa [18], and the introduction to the “6th International Conference on Precision Machinery and Manufacturing Technology” [19]. It is worth noting that none of the identified papers dealt with ceramic-based AM.

Naturally, this was a very rough attempt to grab a general picture of the state of the art, and relevant papers may have remained out of sight because of indexing with different keywords. Furthermore, as exemplified in Table 1 [4,10,11,12,13,14,16,17,20,21,22,23,24,25,26,27,28,29,30,31], the wide range of different terms used as keywords in published papers makes it difficult to conduct an exhaustive bibliographic search.

On the other hand, research in the field is also flourishing outside the archival literature. Chen et al. [22] filed 15 “security tools” for AM that were already available in the marketplace in 2019. The list completed by Chen et al. [22] included both “virtual security tools”, to be integrated in the digital downstream at the network or software level, and “physical security tools”, to be embedded as a fingerprint in the real AM object. In addition, anti-scanning coatings were mentioned in order to deter reverse engineering [32,33]. However, the scarcity of the existing body of literature with respect to the strategic importance of traceability to foster the advancement of AM suggests that this field of research will experience a substantial growth in the near future.

### 2.3. Basic Requirements of Tagging Features in AM

The survey of the archival literature points out that, to be effective, a tagging feature should fulfil some basic requirements regardless of the specific AM technique in use:

- The tag should be tamper-resistant;
- The tag should be copy-resistant;
- The tag should be tear- and wear-resistant;
- The tag should be unobtrusive, meaning that it should fit the size and shape of the object;
- The tag should be compatible with the AM process in use, meaning that its implementation should easily integrate into the printing workflow. For sensors and detectors, which must be embedded into the part, the integration procedure should not interfere with the printing hardware and normal operations; for embedded structural features, ideally the tag should be printable with the part itself, otherwise its implementation should require minimal additional steps;
- The tag should be compatible with the part’s intended usage; for example, deterministic marks based on pores or local density alterations should not undermine the structural reliability of load-bearing components, whereas chemical fingerprints should not compromise the biocompatibility of biomedical devices;
- The tag should have a minimal impact on the part’s cost, as only few parts would have an added value high enough to justify the cost increase;
- If identification is required and not just authentication, the tag should be universally unique; otherwise, the tag should support namespacing. In computing, a namespace is a set of signs that are applied to identify and refer to objects of various kinds. As an example, in hierarchical file systems, files are grouped in directories, where each directory is a separate namespace. Even if the same name is attributed to two files in two different directories, the files remain uniquely identified because they belong to different namespaces;
- The tag should be “detectable”, which means that its presence should be easily and unambiguously revealed with an appropriate detector. For instance, the presence of a unique combination of chemical elements embedded as a chemical fingerprint can be detected with an X-ray spectrophotometer. It is worth noting that, in this example, the tagging feature is a precise mix of specific elements in specific weight fractions and, therefore, as an integral part of the detection process, the detector should be able to measure the relative amounts of such elements. Basically, “detection” is responsible for conveying a minimum amount of information: “Yes” or “No”, namely, “yes, the tag is present” as opposed to “no, the tag is not present”. Interestingly, “detectability” does not imply “visibility”, as long as anti-counterfeiting marks can be detectable with an appropriate probing system but hidden from sight for an additional level of security. In principle, if the same tagging feature is common to all the products from the same producer, detection can suffice for authentication; if the tagging feature is unique to each product, detection can suffice for identification;
- Although, strictly speaking, this is not required for authentication and identification, tags may be “readable”. In particular, this is the case of deterministic tagging features that encode a precise message, for example, the QR code that contains the serial number of a product. In order to read a deterministic tag, the detector should be coupled with a program that converts the acquired data to structured “bits” of information. In this way, the “tag detector” becomes a “tag reader” and both the tag reader and the associated de-coding program must be trusted to correctly read and interpret the tagging feature. Interestingly, the operator that receives the product (which, in different scenarios, may be the end user, the customer, or an intermediate actor along the supply chain) has in mind an expected manufacturer and, accordingly, makes a guess of the appropriate verification method to detect and read the expected tag from that manufacturer.

### 2.4. How to Tag?

A comprehensive strategy for securely identifying and authenticating AM products needs a well-concerted combination of legal measures to protect the technical and intellectual properties, preventive measures to discourage the customer from knowingly buying a fake product, informative measures to guide the client through the purchase, and technical measures to allow for authentication or identification [34,35].

The technical measures account for the implementation of a “tagging feature” and for the development of an informatic technology to store the identification data associated with the tagging feature [36]. Whilst the authentication and, even more so, the unique identification of AM parts is obviously a cross-disciplinary task, the implementation of the tagging feature is the key step where materials engineering comes into play on account of the complicated and interlaced correspondence existing between feedstock materials and 3D printing hardware [37].

As discussed in the following sections, AM parts can be tagged by two different methods:

- Introduction of detectors: a mechatronic component (for example, a radio frequency identification (RFID) chip) that is integrated within the printed part or placed on its surface; whereas standard radio frequency (RF) tags have no identification capability, RFID tags transmit a signal that carries a code to identify it from a multitude of other tags;
- Introduction of embedded “structural” features: the AM process is used to provide the part with a tag that is integral to the structure of the part itself such as a geometric mark (for example, a barcode or a QR code), a chemical fingerprint or a random distribution of “spots” (for instance, pores, impurities or optical markers).

## 3. Sensors and Detectors

Integrating electronic systems in AM parts introduces new functionalities in components with complex or customised geometries (“integrated electronics”) [38]. Sensors may include thermocouples, piezo-sensors, fibre optical sensors, strain gauges as well as detectors such as RFID chips. To some extent, RFID chips are similar to barcodes and QR codes in that they are currently applied to tag and categorize clothes, shoes, vehicles, animals and even humans. However, unlike barcodes and QR codes (which are “read-only” systems), RFID chips use electromagnetic fields that can be detected with a scanner or a reader and, therefore, they are designed for wireless, contactless, two-way communication of information over relatively short distances.

In principle, RFID systems are able to send signals even through highly dense materials and, thus, they are of great interest for tagging AM parts. However, for metal-based AM, especially in powder bed fusion (PBF) techniques [39], this point is still the subject of open research. In fact, also depending on their specific composition, metals have a strong shielding effect that interferes with the communication between reader and transponder. As a consequence, according to the literature, the metal shell that surrounds the transponder should not be closed completely, leaving at least a dielectric gap. On the other hand, experiments at the Fraunhofer Institute for Manufacturing Technology and Advanced Materials [16] have proved that the readability of a transponder to be completely embedded in a metal part depends on the penetration depth of the signal, which can be estimated from the characteristics of the signal and from the properties of the embedding material. The research conducted by Paz et al. [16] has thus led to the conclusion that embedding low-frequency transponders is actually feasible, even if the metal shell around the transponder is completely sealed without any dielectric gap.

However, the samples presented by Paz et al. [16] also demonstrated the technical difficulties that arise when a cavity must be embedded in a part produced by laser-based PBF (L-PBF). When printing the test samples (including cylinders and prisms with a rounded side) with a nickel-based alloy (EOS IN718) powder having magnetic and electric conductivity properties similar to surgical steel, Paz et al. [16] observed that the reading of transponders in the high-frequency range required very thin walls (0.15 mm), which are barely feasible with the available L-PBF equipment. The top surface of the shell was particularly troublesome to print, as it is an overhang, but supports could not be introduced because otherwise they would remain entrapped within. As a consequence, defects and incomplete powder fusion were occasionally observed on the top surface, especially in cavities with very thin walls. Moreover, thin-walled samples were prone to thermally induced deformation and required the addition of lateral supports to stabilise their geometry.

Although more secure from damage and tampering, it should be noted that, if completely sealed within the part, a sensor or detector cannot be reached anymore for maintenance or for recharging, even if a potential solution may be electrical induction (wireless) battery charging. Provided they are not damaged, corroded or broken, passive RFID tags can have a lifetime in excess of 20 years, whereas the lifetime of active RFID tags is limited by the power source (usually an internal battery).

**Figure 2:**
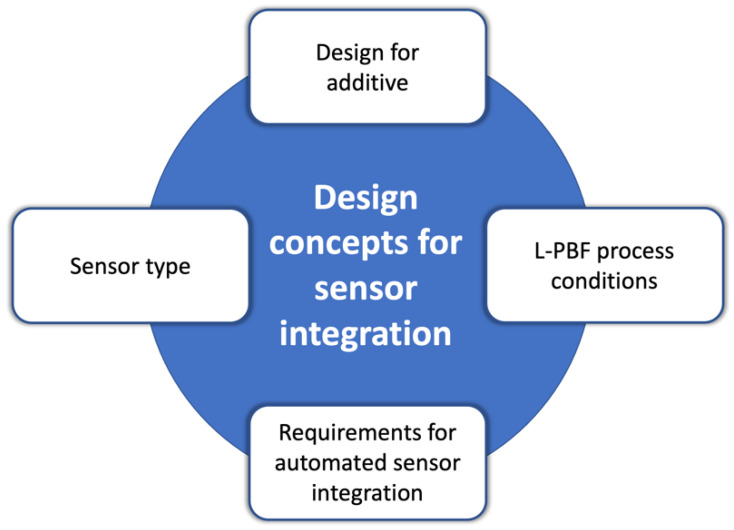
Influences on design concepts for sensor integration into metal L-PBF parts. Adapted from Reference [11], Binder et al. *Procedia CIRP* **2019**, *81*, 992–997, https://doi:10.1016/j.procir.2019.03.240.

As shown in Figure 2, Binder et al. [11] stressed that the automatic integration of sensors in L-PFB parts should take into account several factors, including the object’s geometry that should be revised according to “design for additive manufacturing” guidelines [40].

In practical terms, according to Binder et al. [11], the general procedure to integrate a detector, such as an RFID chip, in L-PBF parts can be schematised in three basic steps:

1. To start printing the part, which has a geometry that includes a cavity to receive the sensor;
2. To interrupt the job to open the cavity by powder removal and to place the tagging device inside the cavity;
3. To complete the printing job.

Placing the mechatronic component upon printing by L-PBF poses several technical issues such as the high temperatures reached during the build-up process, the flow and pressure of inert gas within the chamber, and the presence of electrically conductive particles that may cause contamination. Further, the integration procedure should interfere as less as possible with the operating areas and components of the L-PBF machine. The part design and the integration procedure should also account for the electrical connection of the sensor to the external devices, if required [11]. If the tag must be completely isolated from the environment, passive transponders should be preferred, because they do not contain a battery and, thus, allow for a longer life span [16].

Whereas conventional identification devices consist of an antenna connected to an integrated circuit, chip-less RFID tags do not rely on an integrated circuit to encode the information. Recently, Terranova et al. [17] produced a chip-less RFID tag for AM parts by 3D printing it with the fused filament fabrication (FFF) (aka fused deposition modelling, FDM) technique. This approach offers numerous advantages, because the new tag leverages a three-dimensional geometry to increase its coding capacity compared to conventional bi-dimensional systems. In addition, all the information is concealed in the inner structure of the chip-less tag, whereas the external shape is a simple cylinder, and this contributes to avoiding eavesdropping during the reading process or information retrieval from a visual inspection [17].

## 4. Embedded “Structural” Features

Geometric marks, such as barcodes and QR codes, are commonly employed in the literature to tag AM parts as summarised in Table 2 and Table 3. Barcodes and QR codes have been in use for decades, and currently they are ubiquitous to identify objects [41]. They are typically printed as adhesive labels and then attached to the item or to its packaging. Although easy to apply, stickers may be readily copied, removed or tampered. The added value in AM is that barcodes and QR codes can be co-printed and embedded in the object’s geometry, which makes them more secure [27]. 

Most of the time, barcodes and QR codes are in plain sight. However, both visible and invisible features are implementable for authentication and identification purposes, although they are directed to different stakeholders (as seen in Figure 1). If the validation has to be performed by the final customer, visible marks are often more practical, because they can be seen (but not necessarily “read”) without using dedicated equipment. However, invisible features that require a specific technology to be detected are more difficult to imitate and are essential for verifying the authenticity or even tracking down a single item such as for legal or regulatory reasons [35]. Further, it should be mentioned that the primary goal of all tagging features is to establish a “reactive protection”, meaning that they allow authentication or identification of a product whenever its verification comes into question. However, embedded tagging features also have a “preventive protection” role, since the imitator knows that such tagging features are both difficult to forge and easy to prove [35]. In this regard, the co-existence of visible and invisible tags may offer additional levels of security.

Whereas codes in cryptography are always deterministic so that a message can be reliably encrypted and deciphered, structural tagging features can be either deterministic or non-deterministic. Deterministic features, such as cryptographic codes, are designed to convey a “message” that can be read and, ultimately, decoded. Understanding the meaning of a deterministic feature can be straightforward, as it often happens with logos and other branding marks and writings, or very arduous, as it happens with cryptograms or with symbols that necessitate a key to be solved. The downside of deterministic tagging features is that they are designed according to logical rules that can be figured out and reproduced. However, a structural tagging feature can also be non-deterministic for the sole purpose of authentication or identification. Non-deterministic tagging features are usually based on random patterns and do not convey logical information. Examples of identification through non-deterministic features are frequent in nature. This is the case, for instance, of DNA profiling and fingerprint or iris recognition [42]. The key advantage of non-deterministic marks with the aim of identification, is that the pattern is completely stochastic and, as such, it cannot be reproduced even if all the variables are known [12]. For example, the presence of pores is almost unavoidable in metal-based AM parts [43,44]. However, the location, shape and size of pores are induced by non-deterministic fluctuations in the manufacturing process and, therefore, they are unique to each AM part, and they cannot be reproduced even if the same processing parameters are repeated identically. Pores represent, therefore, a non-deterministic structural feature that can be used for tagging purposes (physical cryptography: the object itself is the tag). However, in order for non-deterministic structural features to be implemented in an efficient identification strategy, it is necessary that enough information can be acquired and stored to enable the recognition of the single object. In fact, the random feature must be readable by some characterisation or detection technique, preferably simple, economical, robust and unambiguous. Moreover, the information must be sufficient and clear enough to disambiguate similar parts [12]. As an additional requirement, the acquired data should be suitable for encoding, storing and integrating into a digital environment for tracking and verification such as a blockchain platform [36]. Another consideration is the readout, whether it be carried out via complex analytical instrumentation in a laboratory, which can be slow, or performed by a dedicated custom reader onsite and in shorter time.

The target of the following paragraphs is to offer an update on the technical measures that have been put in place to provide AM parts with an embedded tagging feature for product authentication and identification. Separate sections will be dedicated to metal parts and to polymer parts. Although arbitrary, this classification mirrors a substantial technological difference, since metals and polymers are typically processed by different methods with direct energy deposition (DED) and PBF being prevalent for metals, and with FFF, PolyJet (InkJet/material jetting) 3D printing and digital light processing (DLP) being prevalent for polymers. Further, on average, polymers are more transparent than metals to most probing methods (e.g., fluorescence spectroscopy), and this dictates the implementation of different tagging strategies. In fact, the same type of tagging feature, such as a barcode or a QR code, can be embedded in different ways in metal parts and in polymer parts. It should be mentioned that, to the best of the authors’ knowledge, as of October 2021, no contributions have been published on specific tagging strategies for ceramic-based AM objects.

### 4.1. Embedded Tagging Features in Metal-Based AM

Both electronic components and embedded structural features are proposed in the literature with the aim of tagging metal-based AM parts. Although less effective than integrated sensors and detectors with the purpose of real-time tracking of items in logistics, embedded tagging features are very practical for authenticating or uniquely identifying AM objects for anti-counterfeiting purposes. For example, tracking codes, such as QR codes, can be directly incorporated into the geometry of AM parts. Chen et al. [13] demonstrated the feasibility of embedding a QR code into single-material AlSi10Mg parts produced by direct metal laser sintering (DMLS). A cube was considered as the test geometry for the part receiving the embedded tag. In order to create the geometry of the QR code, the metal particles in the “empty” regions of the code were not fused and left as loose powder. The residual porosity in the un-fused areas led to a substantial difference in density compared to the surrounding fully solidified metal, which made image acquisition by micro-computed tomography (micro-CT) and processing straightforward. Similarly, in order to tackle the traceability issue of titanium (Ti-6Al-4V) parts produced by L-PBF for biomedical applications, Matvieieva et al. [4] developed a 1D-pharmacode, where the bars of the code were cavities filled with non-molten metal powder. The test specimens were rectangular coupons measuring 40 × 16 × 2.8 mm^3^, whilst the tag’s size was 30.8 × 6 × 0.5 mm^3^. The code was hidden under the skin at a depth of 0.3 mm from the top surface. A comparison among different non-destructive probing methods, including eddy currents, ultrasonic testing, and micro-CT, proved that the micro-CT image had the highest resolution and contrast. However, as pointed out by Matvieieva et al. [4], after being implanted, the parts should be interrogated by means of conventional X-ray methods, which may decrease acquisition contrast and resolution.

Generally speaking, in single-material L-PBF parts, the empty regions of the tagging feature must be created either as a set of voids partially filled with loose powder, if the tag is located inside the solid metal, or as a set of blind holes if the tag is located on the surface of the internal cavity of hollow components. In both cases, the voids in the tracking code are likely to cause a local stress concentration with negative consequences on the static strength and on the fatigue resistance of the printed part [10]. If printed on the external surface, the tagging features may be altered or even removed by post-processing treatments.

Wei et al. [10] proposed to tackle the challenge of code-related voids in single-material parts by shifting to multi-material printing. In this case, a second metal is added to fill the voids in the building material and works as the tagging material. For the multi-material approach to be effective, the constituent phases (namely, building material and tagging material) should meet two basic requirements:

- They should be metallurgically bonded well upon processing (=the two metals should have a low liquid-phase contact angle)
- They should be easily distinguished by some handy detection technique, such as infrared spectroscopy, X-ray fluorescence or X-ray imaging (=the two metals should have markedly different compositions, thermal properties or densities)

In order to print multi-material features, the manufacturing technique should be suitable to control the local distribution of different materials within the same job. DED systems are well-suited to handle multi-material printing and, therefore, they are frequently investigated as “model” processing methods to embed multi-material structural tagging features. For example, laser-based DED (L-DED) has been applied to embed an X-ray fluorescence-responsive molybdenum-based tag in titanium alloy parts [14,45]. In the contribution by Flank et al. [14], a small spot of the molybdenum-based tagging material was printed under the skin or even mixed invisibly into the surface layer of the titanium alloy (Ti-6Al-4V) parts. In both cases, the tagging feature was completely concealed from sight and, at the same time, the absence of voids helped to preserve the structural integrity of the printed parts. Further, the strategy of printing just a small spot of the tagging material instead of mixing a ubiquitous dopant in the whole feedstock powder offered additional options such as different tagging points within the same part geometry. When tested with a desktop X-ray fluorescence detector, the taggant on the surface could be easily located, even if the operator had no prior knowledge of its positioning. The taggant on the surface was invisible to the naked eye but clearly detectable by XRF. However, the spots of taggant became difficult or even impossible to differentiate when hidden beneath 250 µm of titanium alloy. Although the testing samples were simple rectangular coupons, in principle, the tagging strategy proposed by Flank et al. [14] could be extended to more complicated geometries thanks to the relatively small size of the mark.

Although capable of multi-material printing, DED systems present two substantial drawbacks. Firstly, since the printing resolution is relatively low, reproducing the small details of deterministic geometric marks (such as in QR codes) can be very challenging. Secondly, the gas flow that delivers the feedstock powder to the melting pool may splash some particles around and cause cross-contamination of building and tagging materials. Compared to DED, PBF techniques enable a superior printing resolution with relatively low risk of powder splashing, but multi-material printing is still in its infancy, especially if a highly localised distribution of diverse materials is required. The University of Manchester has developed a proprietary ultrasonic selective powder delivery system integrated into an L-PBF device that is capable of distributing different materials within the same job. Basically, a single-layer powder vacuum remover is implemented to locally remove loose particles of the building material, and an ultrasonic dry-powder dispenser is set up to selectively deposit the tagging material where it is needed [10]. Wei et al. [10] applied this technology to embed a multi-material QR code, where Cu10Sn was selected as the tagging material for 316 L stainless steel due to their good liquid-state wettability and diverse properties in terms of composition, thermal conductivity and X-ray absorbance. The QR codes in these samples were built on a 15 mm thick substrate and then progressively covered by 316 L layers. The SEM inspection confirmed the sound metallurgical bonding between Cu10Sn areas and surrounding 316 L matrix. However, some micropores were present at the interface between the Cu10Sn details and the 316 L top layers. The porosity was estimated to be 0.33%, with a mean size of 0.56 µm. The development of micropores at the interface was attributed to the surface roughness of the Cu10Sn areas, whose asperities were not completely healed by the subsequent deposition of the 316 L top layers. The energy dispersion spectroscopy (EDS) analysis confirmed that no cross-contamination had occurred upon printing.

**Figure 3:**
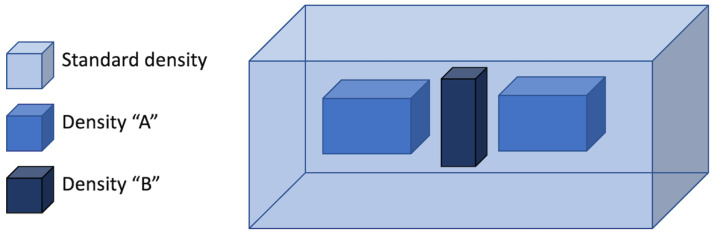
Examples of macroscale deterministic coding obtained by creating domains with different sizes, shapes and densities in a part produced by L-PBF. The different levels of porosity within each area are controlled through the laser energy density as proposed by Eisenbarth et al. [12].

The approaches presented by Chen et al. [13] and by Wei et al. [10] are both based on QR codes, which are deterministic features. However, non-deterministic features have also been explored for the purpose of authenticating and identifying metal-based AM parts. Eisenbarth et al. [12] combined deterministic and non-deterministic codes in metal parts by means of controlled and random process variations. As for L-PBF, the approach proposed by Eisenbarth et al. [12] was based on the assumption that certain combinations of processing parameters cause an irregular and non-predictable track shape and, hence, a random distribution of defects. However, the (average) degree of porosity can be controlled through the volumetric energy density, *E*, which is defined as [46]:

(1) $$E=\frac{P}{v\;d\;l}$$

where *P* is the power of the laser beam, *v* is the scanning speed, *d* is the hatch distance, and *l* is the layer thickness. According to preliminary tests, the density of 316 L parts (rectangular coupons) dropped as soon as the energy density decreased below 70 J/mm^3^. Eisenbarth et al. [12] proved that a purposeful variation of energy density in selected areas leads to the formation of domains having a specific shape where the material properties, particularly the density, deviate from the standard as shown in Figure 3.

In principle, these domains can be shaped to reproduce an assigned geometry, for example, an identification mark. However, as stressed by Eisenbarth et al. [12], the local density should be high enough to preserve the load-bearing capacity of the printed part, since the mechanical properties of the porous domains are certainly lower with respect to a fully dense material and are likely to show high fluctuation due to the inhomogeneous and stochastic porous structure. As to the L-DED parts, instead, Eisenbarth et al. [12] processed rectangular coupons where a slightly paramagnetic material coating (i.e., austenitic steel) was deposited atop a magnetically soft base material (i.e., low carbon steel). The depth of the melt pool, the degree of dilution, and the heat-affected zone in the base material were changed within specific domains by altering the laser power and scanning speed point by point. As a result, the magnetically soft steel was locally mixed with the paramagnetic steel in the melt pool, generating a microstructure with varying magnetic properties, as the two intermixed materials randomly solidified in the melt pool. 

Interestingly, in both AM techniques, the tagging features were non-deterministic at the microscale, because the microstructure derived from the irregular melt pool dynamics, but they were deterministic at the macroscale, since the geometry of the domains with altered materials properties could be controlled through the processing parameters. Nonetheless, in principle, the tagging features could be made non-deterministic also at the macroscale if random generators were applied to design the geometry of the mark or to plan the tool path of the laser.

Eisenbarth et al. [12] tested the codes with an eddy current reading device. This is a very practical method, but the codes should be located in an accessible region of the part near the surface in order to be readable. For the L-PBF parts, eddy current reading was not fully effective to outline the geometry of the porous domains, and this suggests that advanced measurement equipment would be required for code detection. Instead, the L-DED approach was demonstrated to be reliable with a certainty of 500 million to one, if a certain appropriate number of measurement points were used, depending on the measurement uncertainty of the eddy current reading device.

Table 2 [4,10,11,12,13,14,16] summarises the main tagging strategies available in the literature regarding metal-based AM parts. Although this is often left implicit, the proposed tagging strategies are mainly directed at “identifying” the printed parts rather than “authenticating” them.

### 4.2. Embedded Tagging Features in Polymer-Based AM

The research on polymer-based AM is mainly directed towards the development of embedded structural features rather than the introduction of electronic components. Both non-deterministic and deterministic approaches have been proposed in the literature as demonstrated by the several examples reported in the following paragraphs.

#### 4.2.1. Non-Deterministic Tagging Features

Elliott et al. [47] and Ivanova et al. [25] proposed the addition of quantum dots (fluorescent inorganic nanoparticles) as a non-deterministic structural strategy to tag thermoset polymer-based components produced by PolyJet 3D printing. Like a normal ink-jet printer for paper can produce multi-colour prints, a PolyJet 3D printer utilises an array of nozzles to print different materials in the same job and even in the same layer. The multi-material printing ability is key to depositing the build material (which is a standard thermoset ink in the contributions by Elliott et al. [47] and by Ivanova et al. [25]) and the tagging material (thermoset ink + quantum dots) simultaneously, so that the security feature can be easily printed and embedded within the part. Placing the tagging feature within the part, as opposed to its surface, enables an additional level of security, since tampering with the code would cause obvious physical damage to the object.

Quantum dots are fluorescent nanoparticles with a size in the 2–15 nm range. They typically absorb ultraviolet (UV) light and re-emit light in the visible spectrum as a response. The colour of the light they emit can be controlled through their size. The fundamental prerequisite for applying quantum dots, or any luminescent marker, as the embedded tagging feature in PolyJet 3D printed objects is the transparency of the cured thermoset ink to both visible and UV light, which is necessary to facilitate both the optical stimulation and emission of the quantum dots within the printed part. Apart from this material-related requirement, the addition of quantum dots also poses several technical challenges upon printing. Elliott et al. [47] preliminarily demonstrated that the presence of quantum dots (CdSe) up to 0.5 wt% does not impair the cohesion, velocity and volume of the jetted droplets of a typical semi-transparent ink (VeroClear) for PolyJet printing. However, quantum dots tend to agglomerate as proved by the uneven fluorescence of single drops of doped resin deposited on a substrate [47]. In order to investigate the agglomeration phenomena, a polymer mixture with 2 wt% of quantum dots was cured under UV light, and the resulting film was observed with an SEM. The diameter of the agglomerates ranged between 1 and 50 µm with an average size of approximately 20 µm [25]. Agglomeration may have pros and cons: on the one hand, agglomeration may help to create random distributions of particle clusters as the unique identifying feature embedded within the printed part; on the other hand, agglomerates may clog the very narrow nozzles of PolyJet printers (typically around 60 µm in diameter). Moreover, quantum dots and photocurable inks absorb UV light in the same range and, therefore, it is expected that the presence of quantum dots may interfere with the rate and depth of photocuring [47]. In order to account for these hurdles, Ivanova et al. [25] had to implement a semi-automated printing process to demonstrate the printability of quantum dots, since they interrupted the job, jetted and cured the quantum dot-modified ink manually in order to obtain prismatic samples and, finally, restarted the printing job to completion. In spite of these technical complications, which are deemed to be solvable by a proper optimisation of the quantum dot size, amount and distribution in the photocurable resin, Ivanova et al. [25] were able to prove that quantum dot loadings of approximately 0.005 wt% are compatible with the PolyJet process and are detectable inside the object with a simple fluorescent microscope. Increasing the filler loading above 0.005 wt% caused the entire part to glow and potentially increased the risk of clogging the nozzles. An additional advantage of working with 0.005 wt% of quantum dots is that at this concentration, quantum dots cannot be seen to the naked eye, which is the ideal condition for obfuscating the tagging feature. The random distribution of quantum dots therefore creates an embedded pattern that is unique to each printed part. In other words, the object itself can be considered as the tagging feature. In more detail, the addition of quantum dots proposed by Ivanova et al. [25] is an example of a physical unclonable function (PUF), since it meets all the key features of PUFs:

- The relationship between input-challenge and output-response is defined via a physical system; this means that the information about the part’s unicity can be inferred from the distribution of quantum dots only if a proper detection method is applied; in this case, a fluorescence microscope operated at the right magnification, since different patterns of fluorescence signals can be detected from the same object under different magnifications;
- The distribution of quantum dots is completely random;
- The distribution of quantum dots in unclonable, since it cannot be reproduced even by the original manufacturer.

#### 4.2.2. Deterministic Tagging Features

Whereas Elliott et al. [47] and Ivanova et al. [25] opted for non-deterministic marks, the AirCode described by Li et al. [28] is a deterministic technique. Its functioning mechanism is similar to the approach proposed by Eisenbarth et al. [12], in that the marking feature consists in a group of air pockets purposely designed and printed under the part’s surface. While these air pockets are invisible to the naked eye because they are placed under the skin, they drastically change how light is scattered after penetrating the part’s surface, because air has different optical properties from the surrounding 3D printing material. The air pockets become detectable using a specific computational imaging method that separates them from the noise and from spurious signals caused by printing artifacts. Li et al. [28] validated the AirCode tagging system in PolyJet 3D printed parts (rectangular coupons). Since the printer in use could not print voids directly, Li et al. [28] had to use a washable support material to print the pockets. After adjusting the thickness and depths of the pockets, the tag could be successfully detected by the imaging system while being unperceivable by the naked eye. However, as remarked by Li et al. [28], a basic assumption of AirCode is that the printed material is homogeneous and semi-transparent. Whereas this hypothesis holds true for most resins processed by PolyJet, it does not apply to other AM methods. For example, FFF typically produces sharp inter-bead and inter-layer interfaces. Since FFF objects are non-homogenous, tagging them with the AirCode system may be unfeasible [28]. An additional limitation of AirCode is that the method is likely to fail if the object is painted after printing and the paint is completely opaque [28]. Another potential issue with AirCode is its impact on the structural reliability of the printed part, which has not been investigated yet. As pointed out by Li et al. [28], a possible way around this consists of replacing the air pockets with another printing material. However, the optical properties of the tagging material and surrounding matrix must be different enough to locally modify light scattering.

Chen et al. [13] put forward a fully deterministic approach based on embedded QR codes. In addition to metal parts produced by DMLS, Chen et al. [13] assessed the printability of QR codes by FFF, using acrylonitrile–butadiene–styrene (ABS) and a water removable support material, and by PolyJet printing, using either a structural resin (VeroClear) and a removable support material (SUP706) or two different structural resins (VeroWhite, VeroBlack). The QR code was extruded on top of a rectangular plate for the FFF demonstrators. As for the PolyJet parts, the QR code was embedded inside a solid cube and sliced into segments to be printed on different layers at various depths through the part. After micro-CT data acquisition and image reconstruction, the QR code sliced in the VeroClear sample could be read, but the image contrast was relatively low. This was because the resin and its support material belonged to the same family of photocurable resins and had a similar density. Conversely, as previously mentioned, the same approach led to very sharp images when the QR code was generated in AlSi10Mg parts by leaving un-fused powder in selected areas when printing by DMLS. Tensile tests on VeroClear coupons presenting a sliced QR code in the gauge section showed that the ultimate tensile strength and modulus changed by 2% and 0.4%, respectively, as compared to non-tagged benchmarks. Such difference was within the standard deviation range and, thus, statistically insignificant. The weight of the VeroClear specimens with and without a QR code was basically the same, with the difference being only 0.05% [13].

As an alternative approach, Chen et al. [21] observed that anti-counterfeiting features can be integrated in the computer-aided design (CAD) file. If printed according to standard processing parameters, such features will produce defective parts. However, if combined with highly specific printing parameters, such features will not affect the printed parts. The main goal of this strategy is to deter the theft of the CAD file for producing illegitimate copies of the original AM component. In fact, the CAD file alone is useless for printing a high-quality part as long as the right printing parameters are also needed as a separate piece of information. Interestingly, this strategy leverages the development of microstructural defects (for example, tessellation-induced pores or faulty fabrication of embedded features) as evidence of printing the part from stolen data.

Chen et al. [22] further elaborated these two methods (tagging the physical object and tagging the CAD file) and combined them to improve the efficacy of the anti-counterfeiting strategy and, at the same time, to provide unique identification by printing two inter-penetrating QR codes. Out of the two inter-penetrating marks, one code reads as “counterfeit”, whereas the other one is the “genuine” code for identification. Both codes are segmented, and each segment can be printed in a different layer to minimise the effect on the mechanical properties of the final object. Some segments can be shared by both codes. The faulty QR code is designed and printed according to standard protocols, so that it can be easily cloned. The authentic QR code requires instead a carefully controlled process to be printed successfully. The time and effort required to identify the correct orientation and the optimal settings that are needed to print the codes represent a deterrent for hackers. In addition, exactly capturing the interpenetrating QR codes with 3D scanners for reverse engineering is very time consuming due to the small size of each segment, the large number of segments that form the QR codes, and the confusing presence of two QR codes at the same time. On account of the small size of the segments, the design of the interpenetrating codes should account for the specifications of the printing technology, especially the printing accuracy. Chen et al. [22] demonstrated the feasibility of this obfuscating strategy by printing interpenetrating QR codes by FFF and by PolyJet 3D printing. The segmented QR codes could be easily integrated into solid parts such as cuboids and domed cylinders. In FFF prototypes, the marks were built with the support material and embedded in ABS; in PolyJet samples, the QR codes were jetted in white photocurable ink and surrounded by a transparent resin. The QR codes embedded in the FFF parts could be easily read with a micro-CT system. The QR codes in the PolyJet samples did not require any special equipment and could be easily photographed from outside, since they had been printed with a coloured resin in a transparent matrix. One of the main challenges with highly fragmented QR codes is that fine details may be blurred due to printing defects or confused with microstructural porosity. Moreover, as remarked by Chen et al. [22], the mechanical properties of the printed part may be negatively affected if the size of the embedded code is comparable to the part’s size. Chen et al. [22] suggested that the effect of a small and segmented code may be negligible in a large part, but additional testing would be required to confirm this hypothesis. 

Instead of using different materials, Kikuchi et al. [27] proposed engraving the QR code in the 3D printed object, thus obtaining a 3D QR code. This approach requires three input variables, namely, (i) the raw QR code, (ii) the B-spline that describes the object’s surface selected for receiving the QR code, and (iii) the coordinates of a point on the surface to represent the exact target position of the QR code. The algorithm developed by Kikuchi et al. [27] allows for the raw QR code to be incorporated into the freeform B-spline surface by grooving, which is obtained by refining the knots of the B-spline and offsetting the surface inwards in those areas that correspond to the black squares of the QR code. After printing, the 3D QR code can be read thanks to the contrast between light and dark regions caused by ambient occlusion. The deeper the grooves that form the black squares of the QR code, the sharper the contrast. Kikuchi et al. [27] provided several examples of the practicality of engraved 3D QR codes, including a flange printed by FFF with neat ABS, a wax perfume bottle and a heart-shaped chocolate model manufactured by silicone moulding starting from FFF prototypes, and a large-scale model of a pharmaceutical tablets. Readability tests were conducted with plastics in seven different colours. The engraved QR code could be easily read with both iOS and Android mobile phones and an increased offset distance was only needed for Android devices when dealing with a yellow background. The procedure described by Kikuchi et al. [27] offers key advantages, since the engraved QR code can be effectively integrated in any freeform surface regardless of its waviness and does not require multi-material printing. Moreover, the tag is wear- and tear-resistant. However, the grooves and pits that form the 3D QR code are in open sight, which makes it difficult to conceal the tag if needed. In addition, the engraving may interfere with the aesthetic requirements of the printed object and even with its cleaning.

**Figure 4:**
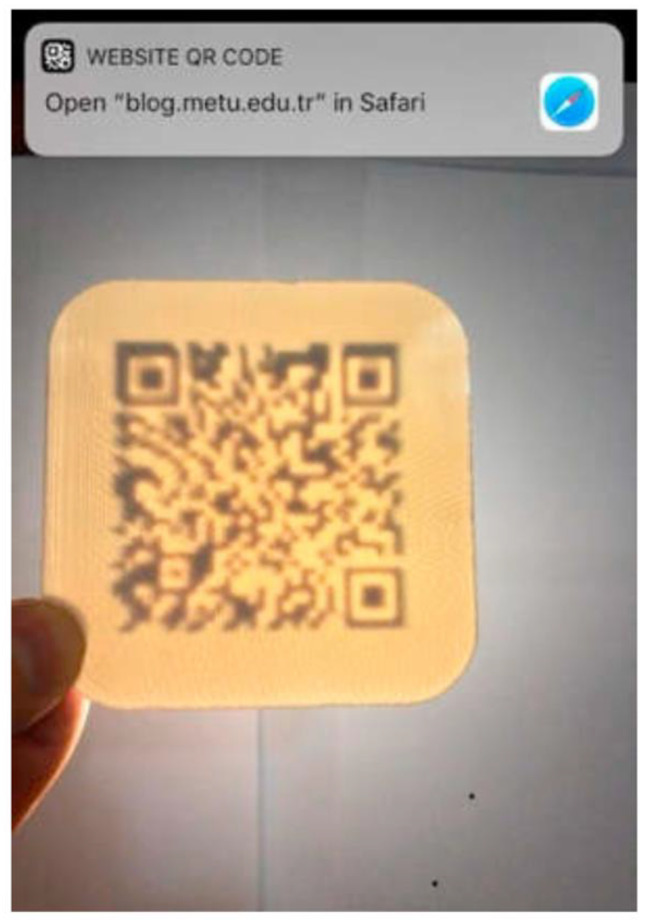
Example of scanning a QR code with background light. Reproduced from Reference [24], Gültekin et al. *Procedia Manuf.* **2019**, *39*, 519–525, https://doi:10.1016/j.promfg.2020.01.411, under the terms of the CC BY-NC-ND license.

Extending the idea of “engraved” QR codes, Gültekin et al. [24] developed an automated approach for embedding the “engraved” QR code on an internal surface of a 3D printed part. The procedure starts with creating the raw QR code according to the input text message and according to the admissible error correction percentage (typically, below 30% to preserve the readability of the code). Then the QR code is integrated into the CAD file. In order to obtain a “negative” geometry, the QR code is moved to the selected internal surface and embedded to a certain depth by a solid difference operation. As a result, the QR code is engraved in the internal surface and, like a watermark in paper, becomes visible from the outside when backlit, which is shown in Figure 4.

**Figure 5:**
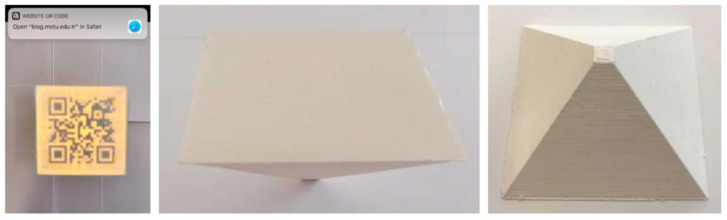
Example of scanning a QR code embedded into a 3D object. Reproduced from Reference [24], Gültekin et al., *Procedia Manuf.* **2019**, *39*, 519–525, https://doi:10.1016/j.promfg.2020.01.411, under the terms of the CC BY-NC-ND license.

Gültekin et al. [24] demonstrated the effectiveness of this approach by FFF, which allowed several geometric shapes to be printed and tagged with an internal QR code as shown in Figure 5. However, as discussed by Gültekin et al. [24], the surface that receives the QR code should not have structural functions. Moreover, reading the code by transmitted light may be difficult with highly opaque materials. 

**Figure 6:**
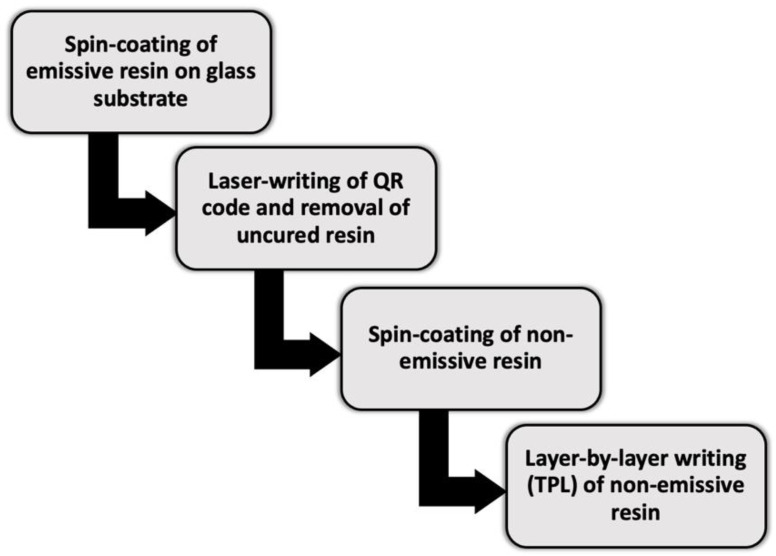
Simplified flowchart of the fabrication process proposed by Jaiswal et al. [26] to produce UV-visible micron-sized QR codes for TPL parts.

Jaiswal et al. [26] demonstrated the feasibility of micron-sized QR codes by two-photon lithography (TPL). To this aim, two different materials were used. The base material for printing was a photo-patternable, non-emissive resin. The base resin was modified by adding 1 wt% of powdered carbon dots (oval-shaped; size distribution in the 2–5 nm range) to obtain an emissive resin visible under UV light. A simplified flowchart of the methodology followed by Jaiswal et al. [26] is reported in Figure 6.

**Figure 7:**
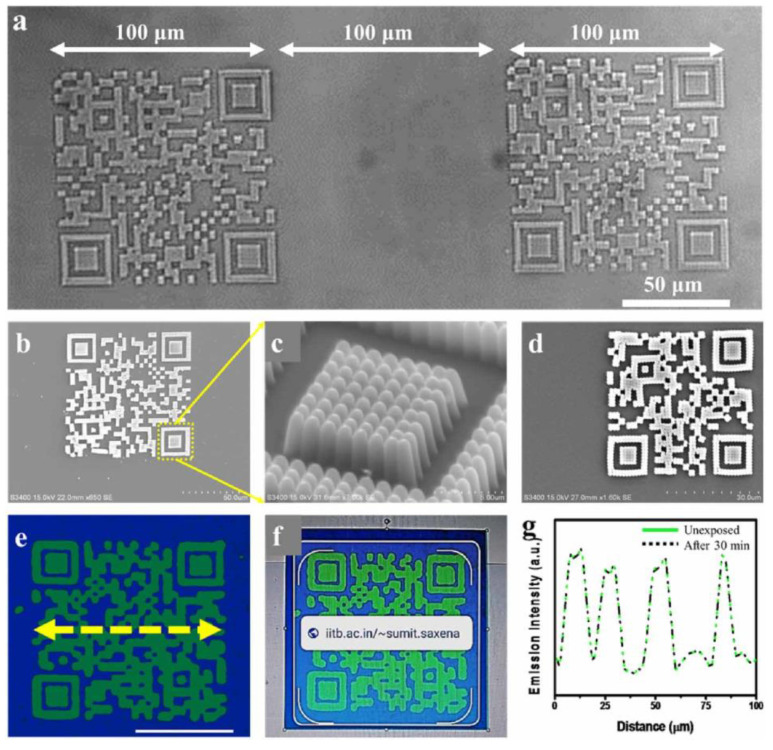
(**a**) Optical image showing two identical QR codes (100 × 100 μm^2^ each), separated by 100 μm, demonstrating reproducibility. (**b**) SEM image showing the top view of the QR code shown in image (**a**). (**c**) Zoomed-in, tilted (30°) view of (**b**), showing the architectural details of the micro-QR code. (**d**) Top view obtained by SEM imaging, showing micro-QR codes fabricated over an area of 50 × 50 μm^2^, demonstrating scalability. (**e**) Fluorescent image obtained from a sample stored under ambient light conditions for a three-month period (scale bar: 50 μm). (**f**) Readout obtained from the image shown in (**e**). (**g**) Average emission intensity profile of (**e**) over a central zone of the QR code (marked with by the yellow line) immediately after UV excitation (green) and after 30 min of continuous irradiation (black, dotted line). Reproduced from Reference [26], Jaiswal et al. *J. Phys. Photonics* **2021**, *3*, 034021, https://doi:10.1088/2515-7647/ac0959, under the terms of the Creative Commons Attribution 4.0 licence.

The first step in the fabrication process required to spin-coat the emissive resin on a glass substrate and to fabricate the QR code using laser-induced two-photon polymerisation. The unexposed (uncured) resin was selectively removed in N,N dimethyl formamide and then a layer of non-emissive resin was spin-coated on top of the QR code to hide and protect it. Lastly, five layers of non-emissive resin were added with layer-by-layer writing in order to make the QR code completely invisible under optical illumination, but still visible under UV light. In addition to concealing the code, the non-emissive resin coat avoided any damage from wear and tear. Two-photon-assisted fabrication offers several advantages, as it is well known for its reproduction fidelity and for its scalability with a voxel size as small as 140 nm for linear structures and approximately 200 nm for more complicated 2D patterns (with a voxel being the building block of structures produced in two-photon polymerisation-assisted fabrication) [26]. Jaiswal et al. [26] demonstrated the accuracy and repeatability of QR codes fabricated over an area of 100 × 100 μm^2^ as exemplified in Figure 7a. A glass coverslip was used as the substrate for demonstrative purposes. The detail in Figure 7c shows that each area of the QR code, in its turn, is composed of sub-microscale features that result from the interaction between ultrashort laser pulses and feedstock material. These fine details work as an additional level of security, because they can be reproduced only if the exact combination of processing parameters is known. According to the analysis conducted by Jaiswal et al. [26], this paves the way for the fabrication of micro-tags, as it is envisaged that the minimum size of the QR code can be scaled down to approximately 4.2 µm for the smallest (and simplest) QR code version with 21 rows and 21 columns. The scalability is illustrated in Figure 7d. However, the process of fabrication described by Jaiswal et al. [26] actually needs to laser-write and develop the QR code before printing the protective non-emissive resin layers, which means introducing additional steps to the standard AM workflow.

**Figure 8:**
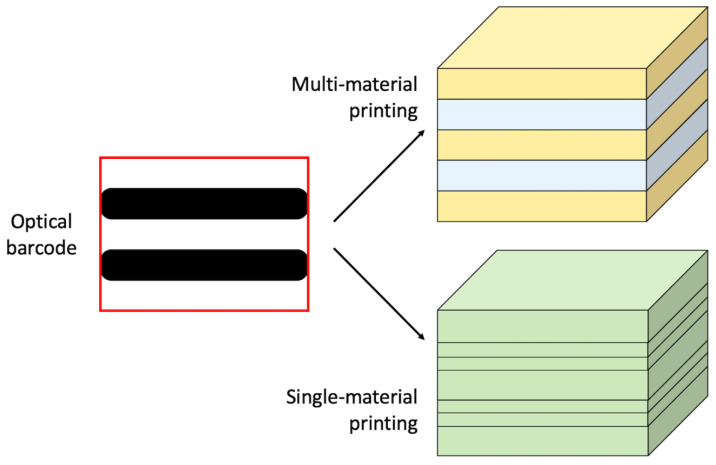
Schematic representation of the LayerCode strategy proposed by Maia et al. [29]. Optical barcodes are translated into 3D printed objects by alternating layers with different materials or layers with variable deposition height (resolution).

The LayerCode tags proposed by Maia et al. [29] are based on the resemblance between the printing layers that form AM parts and the lines that form optical barcodes. However, in order to translate optical barcodes into 3D printed objects, it is necessary to have two different types of layers that correspond to the black and white bars of optical barcodes as exemplified in Figure 8. This is relatively straightforward with multi-material printing technologies, such as FFF and PolyJet, where two different materials can be used to make distinctive layers. The easiest option consists of printing the part with two contrasting colours, but this may pose severe limitations to the aesthetic appearance of the object. Alternatively, the colour can be the same, but the second material can be modified, for example, with a chemical fingerprint or with particles that become visible under specific illumination such as UV and near infra-red (NIR) light. Producing two different types of printing layers is more challenging with machines that are not capable of multi-material printing. To tackle this issue, the G-code can be rewritten and the deposition height (resolution) can be changed during printing to indicate different layer types. For example, a small deposition height (high resolution) can be set to print the layers corresponding to the black bars of the optical barcode, whilst a large deposition height (low resolution) can be applied for the white bars. The complicated geometry of 3D printed parts is another potential issue, since the layered structure may appear curved, deformed or shadowed when imaged with a camera, thus jeopardising the de-coding operation. However, the printing layers run across the whole part and, therefore, the LayerCode tag can be seen along many surface paths as proved by the wide range of geometries considered by Maia et al. [29] including, for example, a figurine reproducing the head of a pharaoh. The new coding and decoding algorithms developed in the framework of LayerCode exploit this redundancy to circumvent the potential imaging ambiguity. The versatility of the LayerCode approach is demonstrated by the numerous examples provided by Maia et al. [29], which include parts printed by FFF, PolyJet and stereolithography (SLA). For multi-material parts, it would be interesting to investigate the effect of the inter-layer bonding strength between dissimilar materials on the mechanical performance. In single-material parts, the layer thickness is the main variable. For example, the mechanical strength of FFF parts along the growth direction is affected by the layer thickness, since inter-layer voids can be minimized by reducing the layer thickness [48]. To some extent, all AM parts are imbued with anisotropy due to the layer-wise build-up strategy and, hence, changing the layering sequence is expected to affect the mechanical behaviour and even necessitate a revision of the part’s design [49,50].

Kennedy et al. [51] pointed out that the identification of the individual object by means of a physical feature, such as the presence of a tag, should be coupled with blockchain technology to maintain and account for the “digital thread” of data associated with the object itself. The digital thread data may include not only the signature information but also additional metadata arising from the part’s design parameters, from the printer code used to control the toolpath and from the printing parameters. Kennedy et al. [51] proposed printing a fluorescent QR code as the link between the physical object and its digital twin on a blockchain platform. To this aim, Kennedy et al. [51] prepared lanthanide–aspartic acid nanoscale coordination polymers (Ln^3+^–Asp NCs)/poly(lactic acid) (PLA) composite filaments for FFF. The new feedstock was formulated taking into account numerous requisites including affordability, suitability for large-volume production and compatibility with standard FFF printers. PLA was chosen as the matrix owing to its ease of printing and widespread usage across many industries. The Ln^3+^–Asp NCs were synthetised according to a simple and non-toxic procedure. The lanthanide species (europium or terbium) was varied in order to tune the fluorescent characteristics. Under TEM, the Ln^3+^–Asp NCs presented irregular and often wire-like morphologies that were smaller than 100 nm in width and 500 nm in length. After verifying the thermal stability of the Ln^3+^–Asp NCs up to 215 °C, composite filaments were produced by solvent casting and extrusion. The filler loading was fixed to 11 wt%, which was necessary to balance the ease of processing (favoured by a low filler loading) and the quality of the readout fluorescence signal (favoured by a high filler loading) of the composite. As proved by the helium ion microscopy inspection, the sub-micron scale size of the coordination polymers was helpful for obtaining composites with a uniform filler distribution, which was crucial for creating a uniform fluorescence emission over large areas. A QR code was then generated to certify the part and printed on a rectangular coupon using a dual-extruder FFF printer with neat PLA as the standard building material. The QR code served as a straightforward identification signature, because its presence could be easily detected through visible fluorescence emission under UV light. The QR code also worked as the link to the blockchain, since its emission was quantified in terms of colour with a common smartphone camera and connected to the corresponding blockchain entry. Interestingly, as stated by Kennedy et al. [51], in this contribution no effort was taken to conceal the QR code that was printed in white on a black background. However, the lanthanide–aspartic acid nanoparticles can be seen under UV light independently of the colour of the polymer matrix in visible light and, therefore, they can be hidden to the naked eye if necessary.

Based on similar luminescence-related physical phenomena, Zhang and Ge [52] developed a functional fibre made of rare-earth luminescent material and fibre-forming polymer as the main raw materials, combined with transparent inorganic pigments and functional additives to enable fibre production via melt-spinning. Different rare-earth luminescent materials have different emission spectra, and even the same rare-earth luminescent material processed under different technological conditions results in different emission spectra. Depending on the nature and concentration of the rare-earth luminescent material, in combination with the other constituent materials and on the melt-spinning parameters, various emission spectral lines are formed that are different from one another when excited with a specific light, and this is why the functional fibres developed by Zhang and Ge [52] have been named “spectrum-fingerprint fibres”. Although not originally addressed to anti-counterfeiting of AM parts, in principle, spectrum-fingerprint fibres might be compounded within a filament for FFF and used to tag the printed part.

Kuang et al. [53] formulated a new two-stage curing hybrid ink to successfully accomplish the fabrication of functionally graded structures by greyscale DLP (g-DLP). The hybrid ink was first partially cured by DLP according to pre-determined greyscale light patterns to form a “green” structure with location-specific properties. Then, the green structure underwent a second-stage thermal curing in order to eliminate most of the residual monomers and to enhance the property gradient. The hybrid ink and related two-stage curing protocol were primarily conceived to obtain functionally graded structures with a very fine tuning of mechanical properties (e.g., stiffness) and thermal properties (e.g., glass transition temperature). However, the position-dependent degree of curing also leads to a position-dependent diffusivity and, hence, to a position-dependent absorption of dyes and fluorescein. This enables the incorporation of marks, such as QR codes, in the printed parts. These microstructural patterns driven by the position-dependent degree of curing are not obvious to the naked eye but become visible after selective diffusion of the dye. In case fluorescein is used instead of a visible dye, the mark is invisible from sight but can be detected under UV light.

In principle, although this topic has not yet been discussed in the open literature, some polymer-based techniques, such as binder jetting and multi-jet fusion, that rely on the localised deposition of tiny droplets of ink or binder, seem well suited to adopt an identification strategy similar to the “machine identification code” (MIC) that was developed by Canon and Xerox in the mid-1980s. The MIC is an example of steganography, which is the “practice of concealing messages or information within other non-secret text or data” (Oxford dictionary online), since it is based on yellow dots (aka tracking dots or secret dots) that certain photocopiers and colour laser printers leave on every printed page to enable identification of the device with which a document was printed. The dots are invisible to the naked eye and, indeed, their existence became public only in 2004 [54]. In fact, the dots when seen in normal daylight are yellow on a white background. In addition, they are extremely small with a diameter of a tenth of millimetre. To spot them out, blue light and magnification are required. The dots are arranged in a dot-matrix that encodes basic information such as the serial number of the printer (which is also a clue to the owner of the device) and the date and time of printing the document [55]. Interestingly, the same matrix is repeated several times across the page for two reasons. Firstly, the redundancy of the information mitigates the consequences of potential local printing errors of the code. Secondly, the matrix can be read even in case only fragments of the page are available [54]. Although the original steganography required to print yellow dots in order to identify the print, other approaches have been put forward such as a local variation in the laser intensity to induce a controlled variation in the shades of grey in the printed text [54]. 

Table 3 [13,21,22,24,25,26,27,28,29,51,53] lists some tagging strategies that have been demonstrated for polymer-based AM techniques.

**Table 3:** Summary of tagging strategies demonstrated in the literature for polymer-based AM parts [13,21,22,24,25,26,27,28,29,51,53].

Reference	AM Technique	Tag	Reading Device
Chen et al., 2017 [21] ^1^	FFF	Features in CAD file	Naked eye (inspection for defects)
Chen et al., 2019 [13]	FFF	QR code, ABS + support material	micro-CT scanner
Chen et al., 2019 [13]	PolyJet	QR code, resin + support material	micro-CT scanner
Chen et al., 2019 [13]	PolyJet	QR code, bi-material	micro-CT scanner
Chen et al., 2019 [22]	FFF	QR code, ABS + support material	micro-CT scanner
Chen et al., 2019 [22]	PolyJet	QR code, bi-material	Digital camera
Gültekin et al., 2019 [24]	FFF	Engraved QR code on internal surface	Phone camera; backlight
Ivanova et al., 2014 [25]	PolyJet	Quantum dots	Fluorescence microscope
Jaiswal et al., 2021 [26]	TPL	Carbon dots	Smartphone (Google Lens); UV lamp
Kennedy et al., 2017 [51]	FFF	Lanthanide–aspartic acid NPs	Handheld UV lamp (+Blockchain)
Kikuchi et al., 2018 [27]	FFF	Engraved QR code on freeform surface	Phone camera; ambient lightning
Kuang et al., 2019 [53]	g-LDP	Selective curing and dye diffusion	Naked eye; UV lamp
Li et al., 2017 [28]	PolyJet	Air pockets	Imaging by light projector and camera
Maia et al., 2019 [29]	PolyJet	Layers with different colours	Digital or iPhone cameras
Maia et al., 2019 [29]	FFF	Layers with variable deposition height	Digital or iPhone cameras
Maia et al., 2019 [29]	SLA	Layers with/without NIR dye	Camera with NIR filter

^1^ Strategy to deter theft of CAD files.

## 5. Discussion and Critical Considerations

### 5.1. Feedstock-Related Specificities: Metal AM vs. Polymer AM

At present, there is no “optimal” tagging strategy suitable for all of AM processes and parts. Different AM techniques rely upon different functional principles, and this calls for the development of dedicated tagging methodologies. For example, embedding “spectrum-fingerprint fibres” [52] may be relatively easy with the filament-like feedstock used in FFF but not with the liquid resins used in SLA and DLP or the micron-sized powders used in PBF. Similarly, optical materials, such as organic molecules or quantum dots, whilst suitable for polymer-based AM, will not survive the processing conditions of laser-based metal AM.

The greatest part of the available literature is still dedicated to the implementation of tagging features in polymer-based AM parts. The prevalence of polymers is due to several reasons. Firstly, AM was formally born with a polymer-based technique in 1987, when 3D Systems Inc. (Rock Hill, SC, USA) first introduced SLA to process photocurable resins [56]. Since then, polymer-based AM has gained momentum and nowadays FFF, selective laser sintering (SLS) and SLA are ranked the most popular 3D printing techniques in both academic and industrial settings [57]. In 2019, the most commonly used material for 3D printing was plastic [58]. Moreover, polymer-based AM methods are relatively inexpensive, since the initial investment costs, the labour and the feedstock materials are less costly than in metal-based systems. The affordability of polymer-based AM techniques fosters their wide adoption in the marketplace and incentivises the research of new materials and hardware solutions. At the same time, the majority of polymer-based AM technologies are compatible with multi-material printing, which naturally facilitates the creation of tagging features. Polymers are transparent or quasi-transparent to many probing methods including X-ray fluorescence and X-ray imaging. On the contrary, the penetration depth in metals is usually very limited [14]. Even more so, several thermoset resins are transparent to the naked eye and allow for a visual detection of embedded tagging features, if required.

At present, the literature offers several examples of organic compounds that can be effectively used in anti-counterfeiting strategies (for example, coordination compounds [18], sequence-coded polyurethanes [59], organic colour-tunable phosphorescent materials [60], polymers with mechanochromic properties and structural colour materials [61,62,63]). However, these materials have not been exploited in AM yet, likely because very little is known about their printability, and extensive research is still needed to unlock their potential. Vice versa, in the future, AM might contribute to the development of more effective tagging strategies in “conventional” polymer processing, for example, enabling the rapid tooling of mould inserts for reproducing personalised QR codes as currently done with soft tooling processes [64].

If metals are considered, generally speaking, tagging DED parts by means of embedded structural features has proved to be easier than tagging PBF parts on account of the multi-material printing ability of DED. However, combining different metals within the same part may cause cross-contamination. Another potential issue, which should be considered, is unintended corrosion caused by the embedded feature. This may be an issue depending on where and how the final part is deployed. For example, if the tag/feature contains metals that are dissimilar to the bulk metal, it presents the possibility of galvanic corrosion occurring should the materials be bridged by an electrolyte such as atmospheric salt spray or bodily fluids, etc. In the case of a bio-implant, whilst part identification may no longer be required after it has been implanted in the patient (unless for forensic reasons), corrosion could undermine the structural integrity of the part, leading to cracks, pitting, etc., and potentially cause the implant to fail.

Since the implementation of selective multi-material printing is still cumbersome with PBF printers and since common tagging approaches based on the controlled creation of porosity may impair the mechanical performance of the printed components, alternative tagging techniques are still needed for PBF parts. For example, microstructure manipulation can be effective for anti-counterfeiting purposes in electron beam additive manufacturing, because the technique enables the site-specific control of the crystallographic grain orientation [65]. However, this approach is still costly and technically complicated to execute. Most of all, very advanced characterisation techniques are necessary to detect the local grain texture and the equipment is not readily available. 

A substantial gap exists in the literature regarding the feasibility of tagging strategies, either by introducing a sensor or detector or by embedding a structural feature, in ceramic-based AM. This is likely due to the difficulty generally experienced when 3D printing ceramic parts as a consequence of their extremely refractory nature. Although new technologies are continuously emerging, ceramic components are often printed starting from a mixture of ceramic powder, binders and other additives that must be debound and sintered to achieve densification [66]. The high temperature treatment is likely to damage thermally sensitive chemical fingerprints and to distort the geometry of deterministic marks.

### 5.2. Microstructural Issues

Theoretically, inserting a sensor or detector does not immediately imply a change in microstructure. However, the part’s geometry must be modified to accommodate the macroscale cavity that will receive the electronic device. This is expected to be detrimental to the mechanical performance, although the location and size of the cavity can be chosen at the design level in such a way as to minimise the impact on the structural reliability. Moreover, Paz et al. [16] clearly demonstrated that printing very thin walls to encase the sensor/detector is a technical challenge in metal AM, with inadequate powder consolidation and warping being likely to occur. In this regard, it would be preferable to process materials that, for a given working frequency (*f*), enable a high penetration depth (*δ*) according to:

(2) $$\delta =\frac{1}{\pi f\mu \sigma }\;$$

where *σ* is the specific conductivity of the material and *μ* the magnetic conductivity (permeability); *μ*, in turn, is composed of the magnetic field constant *μ*_0_ and the relative permeability *μ_r_* [16].

Conversely, embedded features always interfere with the part’s microstructure. However, it is not always easy to define the extent of such interference. A main hurdle is that, quite often, published papers are dedicated to explaining the functioning mechanisms and to verifying the detectability/readability of the tag, rather than to exploring the details of the microstructure. Additional research is certainly needed in this regard. Depending on the nature of the tagging feature, the microstructural alteration may be global (for example, the whole part becomes the tagging feature as it happens with the LayerCode system developed by Maia et al. [29]) or local (for example, where a QR code is introduced).

As for local tags, several strategies are followed in the literature to mitigate the impact on the part’s microstructure. A possible solution relies on the miniaturisation of the tag, as the global effect is expected to become irrelevant as the size of the tagging feature becomes negligible with respect to the part’s size [13]. Another advantage of very small tags is that they can suit a wider range of geometries. However, there are technical constraints to this approach, since not all AM methods have the same printing accuracy. For example, as previously mentioned, Jaiswal et al. [26] calculated that the smallest QR code (21 rows and 21 columns) that can be written by TPL has an area of 4.2 × 4.2 μm^2^. Chen et al. [13] reported that the minimum size of a printable QR code (seemingly 25 rows and 25 columns according to graphical data) in their experiments was 42 × 42 mm^2^ for FFF (ABS + support filament), 7.7 × 7.7 mm^2^ for DSML (AlSi10Mg) and 3.8 × 3.8 mm^2^ for PolyJet (VeroWhite + VeroBlack resin). However, the minimum size of the QR code achievable by PolyJet increased to 4.54 × 4.54 mm^2^ when the VeroClear resin with its support material were used as feedstock material. Similarly, tags with very fine details may become blurred during printing and, hence, too noisy to be decoded [28]. Instead of downscaling the tag, Chen et al. [13,22] fragmented the QR code and distributed the segments on different layers. Although this segmentation strategy is useful for minimising the effect of the QR code on the mechanical properties of the printed part [13] and for increasing the security level of the tagging measure [22], detecting the individual segments across the bulk material may be challenging.

Another possible solution relies on the choice of the tagging material. In order for the mark to be detectable, one or more properties (for example, optical, physical and thermal) of the tagging material must deviate from those of the surrounding material. However, the stronger the dissimilarity, the stronger the perturbation on the microstructure and the local stress concentration. For this reason, quite often the tagging material is produced on purpose starting from the base feedstock and adding the minimum amount of functional filler that is necessary to embed a measurable property for detection purposes [51]. This approach is helpful to minimise the mismatch between the material domains that constitute the tag and the surrounding structural material. However, formulating a printable composite feedstock may be arduous, because the addition of the filler modifies the processability of the matrix material. Unwanted reactions may occur between the filler and the matrix, and the filler itself may be degraded by the printing process. Also, this strategy implies that a bespoke tagging material should be formulated for each printing material, which seems to be impractical. In this regard, economic considerations become critical, with common industrial materials (for example, PLA for FFF [51]) or high-end-use applications (for example, medical devices [4,16]) being likely to receive most of the attention.

Sometimes, the tag is left empty, as it happens with the AirCode system described by Li et al. [28]. This contributes to achieving a lightweight structure, but the location of the mark should not interfere with the part’s load bearing function. Another hurdle with empty tags is that not all AM technologies allow voids to be printed directly. Support materials can be used for that purpose but removing them may be cumbersome due to the inaccessibility of closed cavities after printing. For example, Li et al. [28] fabricated their AirCode prototypes by PolyJet printing and, since this technology is incapable of creating void geometries, they printed the top and bottom layers separately, washed away the support and applied cylindrical connectors for assembly.

Regardless of the specific AM technique in use, as a rule, QR codes and other deterministic tags (e.g., barcodes, writings, symbols and sensors) should not be co-located with load-bearing areas [24]. The most appropriate location for them should be analysed on a case-by-case basis, especially for light-weighted components and lattices.

Finally, it is worth mentioning that the introduction of geometric tagging features is incompatible with any post-processing treatment, such as hot isostatic pressing, that may distort or even delete the mark.

### 5.3. Open Challenges and Growth Directions

Presently, deterministic tagging features, such as QR codes, are predominant within the literature. Their success largely depends on the ability to convey a message, which can be a piece of information regarding the printed part or a link to digitalised data. In addition to authentication and identification, QR codes and other deterministic tags may store details about the mechanical performance and other relevant properties of printed parts. This may contribute substantially to the advancement of quality assurance in AM.

However, a potential shortcoming of deterministic marks is that they must be integrated into the part at the CAD level and, therefore, they are not able to avoid counterfeiting in case the digital design file is copied or stolen, unless additional measures are put in place to protect the file itself [21,22,30]. This is a critical issue for AM that is often defined as “digital manufacturing”, because it relies on a workflow that is mostly digital until printing [13]. In fact, the action of physically printing the object is the last step in a long process chain including modelling and drawing with CAD tools or with scanning- and tomography-based technologies, conversion to .stl (or another AM-oriented format) file, slicing, generation of the G-code that actually controls the printer, and several additional steps that are specific to different AM techniques [31]. Since geometric marks, such as barcodes and QR codes, are introduced at the drawing level, they are vulnerable through all the subsequent digital steps [13].

Another issue related to deterministic marks being introduced at the drawing level is that the CAD file must be modified to change the tag. If provenance assurance is needed for a batch of identical parts, repeatedly reproducing the same mark on all of them may be a sensible approach. However, if single items must be identified for traceability and quality assurance, the tag should be different for each part. To this aim, the tagging feature must be individually created and integrated into the part’s CAD file. Then, the CAD file must be processed, the new geometry must be sliced, and the G-code generated again according to the updated model. Although overlooked in the literature, this point is actually very important, because if the tagging feature could be modified directly at the printing level, the digital manufacturing steps would remain unchanged with a substantial gain in terms of processing time and cost. At present, as they are described in the literature, deterministic tagging measures are not very well suited to accommodate a change in the tag at the printing level. However, an interesting solution may come from the combination of non-deterministic microstructural features with deterministic macroscale marks as proposed by Eisenbarth et al. [12]. Even if the overall geometry of the tag is always the same, the microstructural details (pores, for example, or stochastic distribution of matter in multi-material melt pools) are unique for each part. In principle, this makes it possible to merge the advantages of deterministic marks, especially the ability to convey a message, with the unique identification capabilities of non-deterministic marks, while by-passing the need to revise the digital manufacturing steps. However, this method does not allow the conveyed message to be updated at the printing level and, therefore, other methods are deemed necessary, for example, if the material properties of the individual part must be certified through the tagging feature.

**Figure 9:**
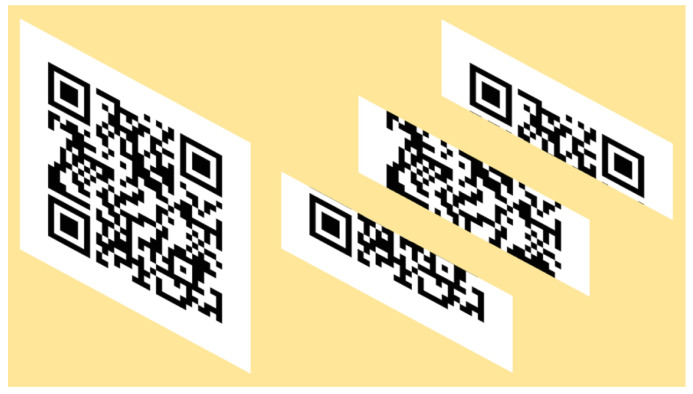
Example of a static QR code split on three different levels. Schematic example of the segmentation strategy proposed by Chen et al. [13] (the static QR code redirects to A. Sola’s ORCID page, https://orcid.org/0000-0002-8649-9388, accessed on 22 December 2021).

Especially if a deterministic mark is based on a geometry that can be easily reproduced or decoded, obfuscation strategies may be required to conceal the tag and prevent information theft upon visual inspection. For example, if a multi-material strategy is adopted to print a code on the part’s surface, the taggant should be indistinguishable with the naked eye from the building material. If the mark is embedded under the skin, a trade-off must be reached, as embedding too close to the surface may not be sufficient for invisibility, whilst embedding too deep may impair detection. In principle, under-the-skin tags can be segmented and spread on different printing layers [13] as shown in Figure 9.

Introducing sensors is key to integrating mechatronic functionalities in AM parts and detectors may be very practical in logistics. However, one of the main drawbacks of this approach is that integrated electronics always require the creation of a cavity, which may be a hindrance to the achievement of the targeted geometry of the printed part. Moreover, the presence of a cavity may reduce the life expectancy of load-bearing components. It is worth noting that, in spite of the progressive miniaturisation in electronics, an analysis of different mechatronic components recently conducted by Binder et al. [11] allowed to define the standardized design space for a generic sensor as a rectangular cavity of 10 × 10 × 5 mm^3^.

Theoretically, provided that an appropriate detector is available, capturing the presence of the tagging feature by itself should be enough for authentication or identification purposes. In this regard, the establishment of a secure strategy to ensuring provenance, quality compliance and intellectual property protection in AM largely depends on existing knowledge and new achievements in materials science and technology. However, as already happens for diamonds and fashion products [67], emerging trends in the literature suggest that, in the future, traceability in AM will also benefit from digital platforms and especially from blockchain-based solutions, where the virtual twin of the physical object is safely stored in the blockchain and the tagging feature becomes the hyperlink between the physical and cyber worlds [20,23,30,51,68,69]. To this aim, materials engineers will be asked to integrate manufacturing technology and information systems.

## 6. Conclusions

The authentication or identification of additive manufacturing (AM) parts is by nature a cross-disciplinary task, since it involves legal, ethical, digital, material and technological issues. In this complicated framework, materials engineers are asked to understand what kind of tagging feature (deterministic or non-deterministic, visible or concealed, localised or distributed) should be preferred for each kind of object and how existing materials and 3D printing hardware should be jointly modified to create such feature. Whereas the body of literature proves that AM parts can be provided with a tagging feature, it is not possible to implement a universal strategy to do so, as different approaches suite different materials and different fabrication workflows. Although the greatest part of the literature is focused on authentication and identification, QR codes and other deterministic marks may also provide information regarding the material properties of the printed part, which would greatly contribute to improving present-day strategies for quality assurance in AM. Moreover, additional efforts should be directed to investigating the effect of introducing a tag on the part’s microstructure and related properties, especially mechanical strength and reliability. In addition, further research is needed to digitalise the tagged object, since the creation of a digital twin on a virtual platform is expected to become increasingly important for tracking, protecting and certifying AM parts.

## Figures and Tables

**Table 1:** Examples of keywords used in published papers regarding traceability of AM parts [4,10,11,12,13,14,16,17,20,21,22,23,24,25,26,27,28,29,30,31]. Keywords are listed exactly as they appear in the original reference.

Reference	Keywords
Alkhader et al., 2020 [20]	Additive manufacturing, blockchain, supply chain, 3D printing, cybersecurity, trust, traceability
Binder et al., 2019 [11]	Additive manufacturing, laser-based powder bed fusion, sensor integration, design concepts, embedded electronics
Chen et al., 2017 [21]	Computer-aided design, additive manufacturing, 3D printing, security, cybersecurity
Chen et al., 2019 [13]	Additive manufacturing, computer-aided design, product authentication, security, 3D printing
Chen et al., 2019 [22]	3D printing, additive manufacturing, anti-counterfeiting, reverse engineering, security
Eisenbarth et al., 2020 [12]	Powder bed fusion, directed energy deposition, coding, anti-counterfeiting, eddy current testing
Flanck et al., 2017 [14]	Metals additive manufacturing, anticounterfeiting, intellectual property protection
Ghimire et al., in press [23]	Industry 4.0, IoT, additive manufacturing, blockchain
Gültekin et al., 2019 [24]	QR code, additive manufacturing, 3D printing, fused filament fabrication
Ivanova et al., 2014 [25]	Additive manufacturing, nanocomposites, quantum dots, cryptograph
Jaiswal et al., 2021 [26]	Additive manufacturing, anti-counterfeiting, two-photon lithography, fluorescence encoding, sub-micron-scale patterned QR code
Kikuchi et al., 2018 [27]	QR code, B-spline surface, 3D printing
Li et al., 2017 [28]	Digital fabrication, 3D printing, unobtrusive tags, air pockets, sensing
Maia et al., 2019 [29]	3D printing, information embedding, fabrication, physical hyperlinks
Matvieieva et al., 2020 [4]	MDR, component identification, traceability, barcode, laser beam melting, powder bed fusion
Paz et al., 2014 [16]	Surgical instruments, additive manufacturing, selective laser melting, RFID chips
Shi et al., 2021 [30]	Additive manufacturing, blockchain, cyber–physical security, encryption, G-code protection
Terranova et al., 2020 [17]	3D printing, additive manufacturing, radio frequency identification (RFID), chip-less RFID, mounted on metal
Wei et al., 2018 [10]	Anti-counterfeiting, additive manufacturing, embedded security features, multiple-material, selective laser melting, QR code, non-destructive inspection
Yampolskiy et al., 2018 [31]	Additive manufacturing, 3D printing, AM security, taxonomy, survey

**Table 2:** Summary of tagging strategies demonstrated in the literature for metal-based AM parts [4,10,11,12,13,14,16].

Reference	AM Technique	Tag	Reading Device
Binder et al., 2019 [11]	L-PBF	RFIDs in AlSi10Mg parts	RFID detector
Chen et al., 2019 [13]	DMLS	QR code, loose powder (AlSi10Mg)	micro-CT scanner
Eisenbarth et al., 2020 [12]	L-PBF	Deterministic shapes with locally deviating material properties in 316 L parts	Eddy current reader
Eisenbarth et al., 2020 [12]	L-DED	Dilution of two materials (i.e., austenitic steel and low carbon steel) with different magnetic permeability	Eddy current reader
Flanck et al., 2017 [14]	L-DED	Selected areas with taggant (i.e., molybdenum) in Ti-6Al-4V parts	X-ray fluorescence spectroscopy
Matvieieva et al., 2020 [4]	L-PBF	1D-pharmacode code, loose powder (i.e., Ti-6Al-4V)	Eddy current reader, ultrasonic reader, micro-CT scanner
Paz et al., 2014 [16]	L-PBF	RFIDs in nickel-based alloy (EOS IN718) parts	RFID detector
Wei et al., 2018 [10]	L-PBF, modified	QR codes with taggant (i.e., Cu10Sn) in 316 L parts	X-ray digital imaging receptor ^1^

^1^ Other used reading techniques but less effective: IR spectral imaging, X-ray fluorescence.

## Data Availability

Not applicable.
